# Group B streptococcal infections in infants in Iceland: clinical and microbiological factors

**DOI:** 10.1099/jmm.0.001426

**Published:** 2021-09-23

**Authors:** Birta Baeringsdottir, Helga Erlendsdottir, Erla Soffia Bjornsdottir, Elisabete R. Martins, Mário Ramirez, Asgeir Haraldsson, Thordur Thorkelsson

**Affiliations:** ^1^​ Faculty of Medicine, University of Iceland, Reykjavik, Iceland; ^2^​ Department of Clinical Microbiology, Landspitali University Hospital, Reykjavik, Iceland; ^3^​ Instituto de Microbiologia, Instituto de Medicina Molecular, Faculdade de Medicina, Universidade de Lisboa, Lisboa, Portugal; ^4^​ The Children’s Hospital, Landspitali University Hospital, Reykjavik, Iceland

**Keywords:** GBS infections, infants, meningitis, neonates, sepsis

## Abstract

**Introduction:**

Group B streptococcus (GBS) is a leading cause of invasive neonatal infections. These have been divided into early-onset disease (EOD; <7 days) and late-onset disease (LOD; 7–89 days), with different GBS clonal complexes (CCs) associated with different disease presentations.

**Hypothesis/Gap Statement:**

Different GBS CCs are associated with timing of infection (EOD or LOD) and clinical presentation (sepsis, meningitis or pneumonia).

**Aim:**

To study infant GBS infections in Iceland from 1975 to 2019. Are specific GBS CCs related to disease presentation? Is CC17 overrepresented in infant GBS infections in Iceland?

**Methodology:**

All culture-confirmed invasive GBS infections in infants (<90 days) in Iceland from 1975 to 2019 were included. Clinical information was gathered from medical records.

**Results:**

A total of 127 invasive GBS infections in infants were diagnosed, but 105 infants were included in the study. Of these, 56 had EOD and 49 had LOD. The incidence of GBS infections declined from 2000 onwards but increased again at the end of the study period. Furthermore, there was a significant increase in LOD over the study period (*P=*0.0001). The most common presenting symptoms were respiratory difficulties and fever and the most common presentation was sepsis alone. Approximately one-third of the cases were caused by GBS CC17 of serotype III with surface protein RIB and pili PI-1+PI-2b or PI-2b. CC17 was significantly associated with LOD (*P*<0.001).

**Conclusion:**

CC17 is a major cause of GBS infection in infants in Iceland. This clone is associated with LOD, which has been increasing in incidence. Because intrapartum antibiotic prophylaxis only prevents EOD, it is important to continue the development of a GBS vaccine in order to prevent LOD infections.

## Abbreviations

ALP2-4, alpha-like proteins 2-4; BCA, C alpha protein; CC, clonal complex; CPS, capsular polysaccharide; EOD, early-onset disease; EPS, epsilon protein; GBS, group B streptococcus; LOD, late-onset disease; MLST, multilocus sequence typing; PCR, polymerase chain reaction; PROM, premature rupture of membranes; RIB, rib protein; ST, sequence type.

## Introduction

*Streptococcus agalactiae* or group B streptococcus (GBS) is a leading cause of invasive neonatal infections [1]. The bacterium colonizes the gastrointestinal and genital tracts of humans [2, 3] and up to 44 % of women carry GBS in the vagina during pregnancy [4–8]. Most commonly, GBS infections of newborns occur because of an ascending infection in pregnancy or the newborn becomes colonized during passage through the birth canal [2]. Approximately half of infants born to women who are carriers of GBS will be colonized, but only 1–2 % of those will develop an invasive GBS infection [2, 3].

Infections in infants have been divided into early-onset disease (EOD) occurring 1–6 days after birth and late-onset disease (LOD) occurring 7–89 days after birth [2, 9]. GBS disease in infants most commonly presents as sepsis, pneumonia or meningitis [1, 2, 9] and studies have shown that meningitis is significantly more common in LOD [9, 10]. The most common risk factors for EOD are known maternal colonization, premature rupture of membranes (PROM), preterm delivery (<37 weeks’ gestation), prolonged rupture of membranes (≥24 h), chorioamnionitis and intrapartum fever (≥38 °C) [2, 3, 11]. More than 80 % of infants with EOD present with respiratory distress, i.e. tachypnea or grunting, with retractions and nasal flaring. Infections can also present as apnea, especially in the preterm infant [2]. Maternal colonization of GBS is also a risk factor for LOD and many infants who get LOD are born preterm [12]. Other risk factors for LOD, such as nosocomial transmission and contaminated breast milk, have been postulated [12]. Clinical signs of LOD infections in infants are often nonspecific [2].

Discussion of preventive measures against GBS infections in infants started around 1990 [13]. Since then many countries have implemented preventive measures, giving intrapartum antibiotic prophylaxis based on risk factors or screening for GBS in pregnancy [1, 14]. Following these measures the incidence of EOD but not LOD has declined [1], and some studies have suggested that the incidence of LOD may be increasing [15, 16]. The incidence of all GBS infections in infants from 0 to 89 days of age is reported to be 0.49–0.53/1000 live births [17, 18].

An important virulence factor for GBS is the capsular polysaccharide (CPS), which has been used for serotyping and understanding the epidemiology of GBS [19]. To date, 10 different types of CPS have been described (Ia, Ib and II–IX) and the most common serotype in neonatal infections is type III [19]. GBS also has three different types of pili and a few types of surface proteins [alpha-like proteins such as C alpha (BCA), rib (RIB), epsilon (EPS) and alpha-like proteins 2–4 (ALP2-4)] that have been identified [20]. In recent years, with more advanced molecular methods such as multilocus sequence typing (MLST), GBS has been categorized into several sequence types (STs) and clonal complexes (CCs) according to genetic relatedness [21].

The aim of this study was to describe GBS infections in infants in Iceland from 1975 to 2019 and determine if there is a relationship between the clinical presentation of GBS infections and microbiological factors of the bacteria, such as serotypes, CCs, surface proteins and pili, and also to determine if clonal complex 17 is overrepresented in infections in Icelandic infants, as has been shown in studies from other countries [22–24].

## Methods

All culture-confirmed invasive GBS infections (from blood or cerebrospinal fluid) in infants (<90 days) in Iceland in the years 1975–2019 were included. The data were accessible from the database of the Department of Clinical Microbiology, Landspitali University Hospital. Clinical information was gathered from infant and maternal medical records using national identification numbers. Information on the following variables related to pregnancy and birth were collected: gestational age, complications during pregnancy, mode of delivery, maternal GBS status, premature rupture of membranes (PROM), maternal fever ≥38 °C during delivery, intrapartum antibiotic prophylaxis and diagnosed chorioamnionitis. The following variables were gathered regarding the infant and clinical presentation of the infection: gender, birth weight, APGAR score, age at diagnosis, first clinical signs of infection, results from blood culture and culture from cerebrospinal fluid, antibiotic treatment, number of days in hospital, diagnosis of sepsis, meningitis and/or pneumonia and deaths.

All available isolates from all GBS infections during the study period were kept frozen at the department. Serotyping had been performed earlier at the Department of Clinical Microbiology, Landspitali University Hospital, Reykjavik, Iceland and at the Faculdade de Medicina, Universidade de Lisboa, Lisbon, Portugal by one of the authors (E. S.B.), as recently published [25, 26]. Capsular serotyping was performed by latex agglutination test [27]. MLST was used to categorize the bacteria into sequence types (STs) [28] and from that into clonal complexes (CCs) using the goeBURST algorithm [21]. A multiplex polymerase chain reaction (PCR) was used to test for the presence of pili and surface protein genes [29].

Statistical analysis and graphical representation were performed using the software Rstudio. The chi-squared test and Fisher’s exact test were used to compare two categorical variables, the two-sample *t*-test was used to compare two means of continuous variables and the Wilcoxon test was used to compare two medians of non-normally distributed continuous variables. Correction for multiple testing was done with the Bonferroni method where appropriate. Poisson regression was used for analysis of the incidence of GBS infections. Information on live births in Iceland was obtained from Statistics Iceland (www.statice.is). Results were considered significant if the *P*-value was <0.05.

The study was approved by The National Bioethics Committee (VSNb2015120015/03.03.) with later adaptations and the medical director of Landspitali, University Hospital.

## Results

The number of invasive GBS infections in infants (<90 days) in Iceland from 1975 to 2019 was 127, and of these 3 infants were reinfected [year 1984 at days 1 (CC17, serotype III, surface protein gene RIB, pili PI-1+PI-2b) and 35 (isolate not available); year 1989 at days 24 (CC17, serotype III, surface protein gene RIB, pili PI-1+PI-2b) and 41 (isolate not available); year 2017 at days 11 and 40 (both isolates CC17, serotype III, surface protein gene RIB, pili PI-2b)]. Of those infections, 107 isolates were available from 106 infants. One medical record was missing (serotype V, CC1). Thus, 105 infants and their isolates were included in the study. There were 60 males (57 %) and 45 (43 %) females. There were 56 (53 %) with EOD and 49 (47 %) with LOD. Information about the infants, pregnancy and delivery, as well as known risk factors for GBS infections, separated into EOD and LOD, is shown in Table 1.

**Table 1.** Information on pregnancy, delivery, GBS risk factors and perinatal clinical factors of 105 infants with GBS infection in Iceland from 1975 to 2019

	Early-onset disease (EOD)	Late-onset disease (LOD)	All cases	*P*-value
	No. (%)	No. (%)	No. (%)	
**No. of cases (%)**	56 (53)	49 (47)	105 (100)	
**Infant**				
Female	19 (34)	26 (53)	45 (43)	0.08
Male	37 (66)	23 (47)	60 (57)	0.08
APGAR 1 min	7 [5–8]*	8 [6–8]*	8 [6–8]*	0.45
APGAR 5 min	9 [7–9]*	9 [8–10]*	9 [7–10]*	0.11
Age at diagnosis (days)	1 [0–1]*	23 [15–53]*	2 [1–21]*	
Deaths	5 (9)	0 (0)	5 (5)	0.06
**Pregnancy and delivery**				
Birthweight (g)	3675 [2998–4083]*	3100 [2123–3837]*	3480 [2573–4030]*	**0.008**
Gestational age (days)	280 [259–286]*	274 [245–283]*	276 [253–286]*	0.19
Twins	2 (4)	7 (14)	9 (9)	0.08
Preterm (<37 weeks)	14 (25)	17 (35)	31 (30)	0.31
Emergency c-section	9 (16)	10 (20)	19 (18)	0.67
Elective c-section	0 (0)	2 (4)	2 (2)	0.21
Assisted delivery	7 (13)	7 (14)	14 (13)	0.78
Intrapartum antibiotics	6 (11)	8 (16)	14 (13)	0.46
Mother diagnosed with chorioamnionitis	5 (9)	1 (2)	6 (6)	0.22
**Known risk factors**				
Mother known GBS carrier	4 (7)	4 (8)	8 (8)	0.73
Premature rupture of membranes (PROM)	29(52)	14 (29)	43 (41)	0.09
Prolonged PROM >24 h before birth	13 (23)	4 (8)	17 (16)	0.09
Intrapartum fever ≥38 °C	10 (18)	3 (6)	13 (12)	0.19

*Median (interquartile range).

Preterm infants were 31 in total (30 %) and out of the 14 preterm infants with EOD, 8 were born after PROM and of these 7 mothers had PROM >24 h before birth. Labour was induced in two mothers, one had high fever and the other had pre-eclampsia. In total, 55 mothers (52 %) had 1 or more risk factors for infection, and of those, 36 (65 %) were mothers of infants who got EOD. In total, 14 mothers received antibiotics intra-partum and of these 6 had infants who got EOD and 8 had infants who got LOD (Table 1). During the study period six mothers with fever ≥38 °C during delivery did not receive antibiotics; five of those cases occurred before 2002.

### Epidemiology

**Fig. 1.**
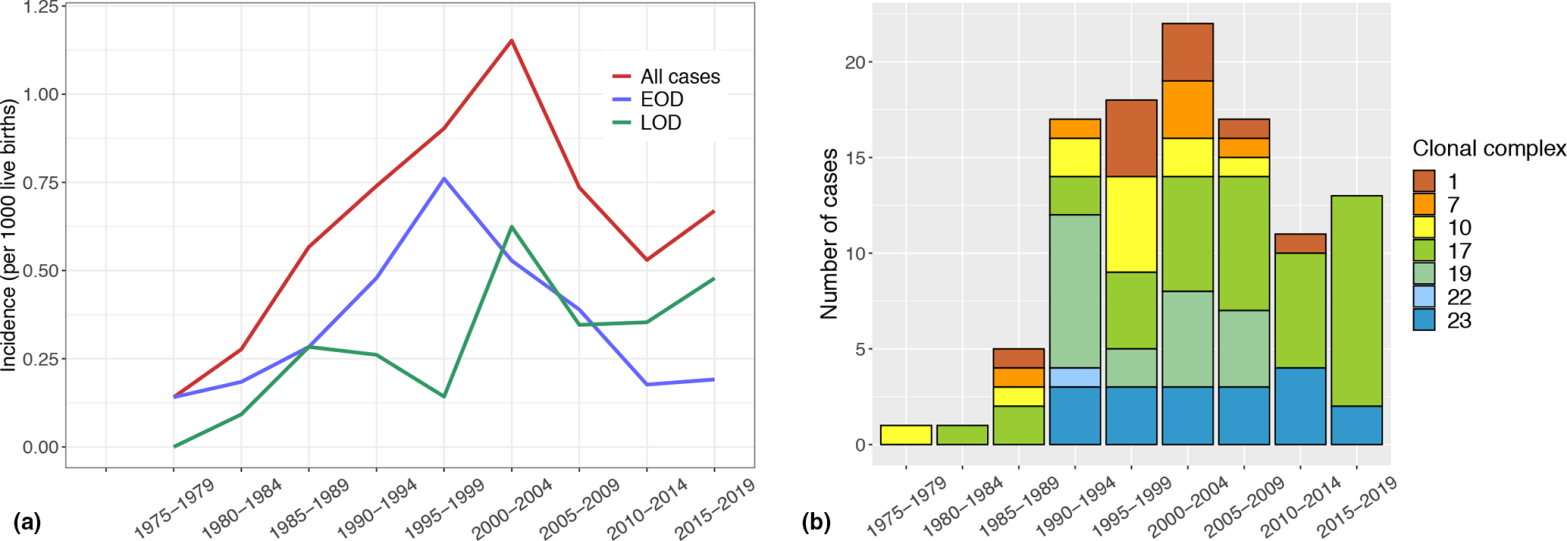
Epidemiology of invasive GBS infections in infants (<90 days old) in Iceland from 1975 to 2019. (a) Incidence of invasive GBS infections in infants from 1975 to 2019 divided by 5-year periods*.* All GBS infections in infants (*n*=124) are included, regardless of the availability of the isolate; three GBS reinfections were excluded. In total, there were 68 cases of EOD and 56 cases of LOD during the study period. (b) Clonal complexes (CCs) of GBS isolates in infants in Iceland from 1975 to 2019 divided by 5-year periods*.* Isolates were of seven different CCs.

The incidence of all invasive GBS infections in infants in Iceland from 1975 to 2019 is shown in Fig. 1a. The incidence increased during the first half of the study period and peaked in 2000–2004 at 1.15/1000 live births. After that, the incidence decreased but increased again at the end of the study period in 2015–2019 to 0.67/1000 live births. The incidence of EOD increased during the first half of the study period and was highest in 1995–1999 at 0.76/1000 live births and decreased after that and at the end of the study period in 2015–2019 the incidence was 0.19/1000 live births. The incidence of LOD increased significantly during the study period (*P*=0.0001) and was highest in 2000–2004 at 0.62/1000 live births. At the end of the study period in 2015–2019 the incidence was 0.48/1000 live births.

### Serotypes and molecular types

The GBS isolates were of six different serotypes, with serotype III being the most common. Of 105 isolates, 56 (53 %) were serotype III, 23 (22 %) serotype Ia, 9 (9 %) serotype Ib, 8 (8 %) serotype V, 5 (5 %) serotype II and 3 (3 %) serotype IV, while 1 was not typable. The GBS isolates were of seven different CCs, with CC17 being the most common. Of 105 isolates, 39 (38 %) were CC17, 19 (18 %) were CC19, 18 (17 %) were CC23, 12 (12 %) were CC10, 9 (9 %) were CC1, 6 (6 %) were CC7 and 1 (1 %) was CC22. Before the turn of the century CC17 comprised <50 % of total isolates. The proportion increased significantly over time (*P*=0.003) and CC17 comprised ~80 % of total isolates in 2015–2019 (Fig. 1b).

Four different types of surface protein genes were found in the isolates in the study. In total, 58 (56 %) had the surface protein gene RIB (*rib*), 21 (20 %) the surface protein gene BCA (*bca*), 20 (19 %) the surface protein gene EPS (*eps*) and 5 (5 %) the surface protein gene ALP3 (*alp3*). The GBS isolates had four different combinations of pili. The most common combination was PI-1+PI-2a among 39 (38 %) isolates, while 35 (34 %) isolates had PI-1+PI-2b, 21 (20 %) isolates had PI-2a and 9 (9 %) isolates had PI-2b. GBS CCs were mostly associated with given serotypes, surface protein genes and pili as previously reported [30]. In total, 18 different lineages of the GBS bacteria were found during the study period. The most common lineage CC17 of serotype III, with the surface protein gene RIB and pili PI-1+PI-2b, was found in 30 infections (29 % of all isolates).

### Clinical presentation

GBS infections in infants were classified as sepsis alone (the most common presentation with 74 infants or 70 %), sepsis with meningitis (16 infants or 15 %), sepsis with pneumonia (13 infants or 12 %) and sepsis with meningitis and pneumonia (2 infants or 2 %). Clinical information on GBS infections and diagnoses of infants separated by EOD and LOD is shown in Table 2. The most common presenting symptoms were respiratory difficulties and fever ≥38 °C. Respiratory difficulties were tachypnea (respiratory rate ≥60/min), grunting, retractions and nasal flaring. Respiratory difficulties were significantly more common in EOD than LOD (*P*=0.001), but fever ≥38 °C was significantly more common in LOD (*P*<0.001). Meningitis was more common in LOD than EOD, although not significantly, and pneumonia was only diagnosed in EOD (Table 2).

**Table 2.** Clinical findings for infants with GBS infections in Iceland from 1975 to 2019 divided by EOD and LOD

	Early-onset disease (EOD)	Late-onset disease (LOD)	All cases	*P*-value
	**No. (%)**	**No. (%)**	**No. (%)**	
**No. of cases (%)**	56 (53)	49 (47)	105 (100)	
Fever ≥38 °C	20 (36)	39 (80)	59 (56)	**<0.001**
Respiratory difficulties	51 (91)	31 (63)	82 (78)	**0.001**
Hospital stay (days)*	10 [8–14]*	11 [9–26]*	10 [8–17]*	0.18
Sepsis	56 (100)	49 (100)	105 (100)	0.49
Meningitis	6 (11)	12 (24)	18 (17)	0.11
Pneumonia	15 (27)	0 (0)	15 (14)	**<0.001**

*Median (interquartile range).

There was a significant relationship between CC10 and pneumonia (*P*=0.03), but it was lost after correction for multiple testing with the Bonferroni method. There was not a significant relationship between CCs and fever or CCs and respiratory difficulties (*P*=0.21 and *P*=0.20). The relationship between presenting symptoms and serotypes, surface proteins and pili was also tested. A significant relationship was found between serotypes and fever ≥38 °C (*P*=0.01), where serotype III caused fever more often and serotypes II and V less often than expected. There was not a significant relationship between presenting symptoms and other factors.

**Fig. 2.**
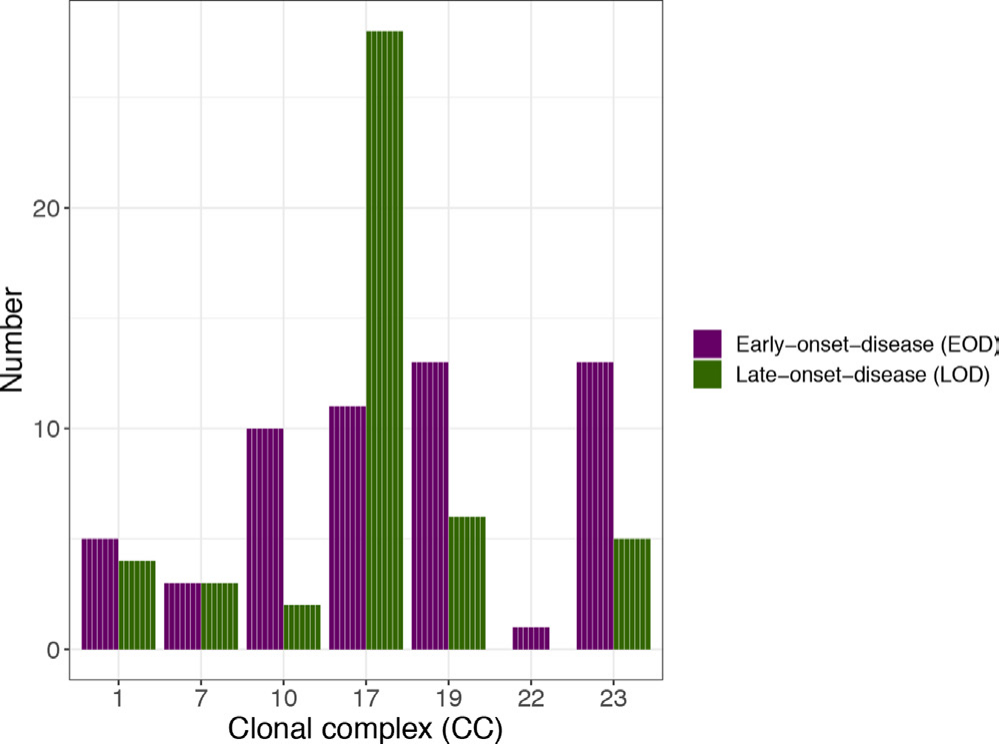
Clonal complexes (CCs) of GBS infections in infants in Iceland from 1975 to 2019 divided by early-onset disease (EOD) and late-onset disease (LOD).

A significant relationship was found between CCs and infection classification as EOD or LOD (*P*=0.002) (Fig. 2). Moreover, CC17 was significantly more common in LOD than EOD (*P*<0.001). CCs 10, 19 and 23 were more common in EOD than LOD, but not significantly (*P*=0.06, *P*=0.25, *P*=0.14).

### Antibiotics

Of 14 mothers who received intrapartum antibiotic prophylaxis, 7 received penicillin (50 %), 4 ampicillin–clavulanic acid (29 %), 1 ampicillin, 1 cefazolin and 1 erythromycin with gentamicin. Ampicillin with either gentamicin or cefotaxime as empirical therapy at presentation was the most common antibiotic used for infants with suspected severe infection (96 infants or 91 %). The most common antibiotic that infants received when GBS infection had been confirmed was ampicillin (98 infants or 93 %). Next were gentamicin (62 infants or 59 %), cefotaxime (40 infants or 38 %), netilmicin (38 infants or 36 %) and penicillin (20 infants or 19 %). When information on susceptibility was available, ampicillin was usually continued alone or changed to penicillin monotherapy.

## Discussion

The incidence of invasive GBS infections in infants in Iceland decreased from a peak in 2000–2004 until it increased again at the end of the study period in 2015–2019. However, when considering fluctuations in the incidence in Iceland, the small size of the population must be taken into account. In accordance with other studies, the incidence of EOD decreased from the turn of the century [1, 31]. This was likely the result of preventive measures against EOD, such as intrapartum antibiotic prophylaxis for women with known risk factors. In agreement with Icelandic studies on GBS infections in infants, covering the years 1976–2015, the incidence of LOD increased significantly during the study period [16, 26]. More studies have shown that the incidence of LOD has remained the same or surprisingly increased in recent decades [1, 12, 15, 31–33]. At the end of the study period in 2015–2019 the incidence of EOD in Iceland was 0.19/1000 live births, slightly lower than the incidence of 0.23 recently reported in the USA [34]. Interestingly, the incidence of LOD in Iceland was 0.48/1000 live births at the end of the period, clearly higher than the incidence of 0.31 in the USA [34]. It seems that the incidence in Iceland is still shifting more towards LOD.

The available isolates were of six different serotypes, with III being the most common. This is in context of a recent meta-analysis of the epidemiology of neonatal GBS infections that included 135 studies showing that serotype III was the most common (61.5%) [18]. In our study the GBS isolates were of seven different CCs and CC17 was clearly the most common. The same has been shown in studies in Sweden and Ireland [22, 23], and in the Netherlands CC17 increased in prevalence during recent decades [24]. In our study, the hypervirulent clone CC17 was also overrepresented and accounted for about 80 % of all GBS isolates from infants in 2015–2019.

The most common presenting symptoms were respiratory difficulties and fever. Respiratory difficulties were significantly more common in EOD than LOD, probably because pneumonia is more often related to EOD. Fever was significantly more common in LOD. This could be because the infection is more advanced at the time of diagnosis or the infant is more mature at that time. The most common presentation in our study was sepsis alone (see Table 2). Meningitis was more common in LOD and pneumonia was only diagnosed in EOD. Other studies have found similar results but with fewer cases of pneumonia in EOD [9, 11, 31]. It is conceivable that pneumonia is related to EOD because neonates may aspirate colonized amniotic fluid or vaginal secretions during the birth process and passage through the birth canal. Meningitis could be more common in LOD than EOD because of later intervention in LOD. Furthermore, infants with LOD had significantly lower birthweight than infants with EOD and a higher proportion of preterm infants, resulting in lower titres of maternal-specific antibodies [35]. Thus, preterm infants could be more susceptible to severe LOD infections.

In this study, CC10 was significantly related to pneumonia but this relationship was lost after correction for multiple testing. To the best of our knowledge, this has not been reported before. However, this must be interpreted with caution because of the few cases and possible coding differences for the diagnosis of pneumonia. This study did not show a relationship between CC17 and meningitis, as other studies have [10, 36]. The proportion of meningitis cases among LOD was 24 versus 66 % in a study from France [10] and only 6 out of 18 cases of meningitis were caused by CC17. It remains uncertain why fewer CC17 meningitis cases occurred in Iceland, but one possibility is that infants received medical care sooner. However, CC17 was associated with LOD, as shown in other studies [22, 36]. A study from Canada on GBS colonization in pregnant women with follow-up after delivery showed that ST17 and ST19 were significantly more likely to persist in vaginal samples than others, despite antibiotic treatment [37]. The CC17 group almost exclusively comprises ST17 [23]. On the same note, all three infants with reinfection in our study had CC17. It can be postulated that the increase in the use of antibiotics during delivery after the turn of the century may have had an impact on the increase in the prevalence of the hypervirulent clone CC17, as other authors have also suggested [38, 39]. The PI-1 and PI-2b pili that most CC17 have seem to be important for adherence to epithelial cells [40]. Furthermore, the PI-2b pilus has been shown to play a role in invasion and possible spread to the central nervous system [41]. In our study there were 39 (37 %) CC17 of serotype III with the surface protein gene RIB, 30 had pili PI-1+PI-2b and 9 only had PI-2b. Thus, this study is consistent with the suggestion that this clone is hypervirulent in neonatal GBS infections.

Risk factors for neonatal GBS infections were confirmed in half of the mothers, and two-thirds of their infants got EOD. PROM >24 h before birth and fever ≥38 °C during delivery, which are known risk factors for EOD [1, 11, 42], were more common in EOD than LOD. Risk factors for LOD are less clear but maternal carriage of GBS and prematurity are two known factors [12]. Preterm infants were 14 (25 %) in the EOD group and 17 (35 %) in the LOD group. Twelve infants with LOD were infected while still in the hospital, suggesting that transmission might have occurred from the hospital environment or from their mothers. Those 12 infants had been admitted for an average of 39 days, 9 were born preterm, 1 mother was a known GBS carrier and 3 mothers received antibiotics intrapartum. Other countries have reported hospital transmissions of GBS [43, 44] and in our study we found three possible cases of hospital transmission.

Fourteen mothers received intrapartum antibiotic prophylaxis. Six had children with EOD and four of them had fever during delivery, which has been correlated with ineffectiveness of antibiotics [45]. Eight mothers had infants with LOD and five infants were infected with CC17, which has been shown to persist in the birth canal [37].

The small number of patients in our study may be regarded as a weakness, but we describe GBS infections in infants from a whole country over several decades.

A recent meta-analysis of 17 studies comparing screening- and risk-based protocols for prevention against neonatal GBS infections showed that screening was associated with lower incidences of EOD [46]. However, the rising incidence of LOD is of concern and other prophylactic measures, such as vaccines, are needed [47, 48]. Given the differences in serotypes and invasiveness in EOD and LOD it is important for every country to follow their epidemiology in the fight against neonatal invasive GBS infections.
